# Early Onset of Tenofovir-Induced Renal Failure: Case Report and Review of the Literature

**DOI:** 10.1100/tsw.2007.164

**Published:** 2007-07-27

**Authors:** Shilpa M. Patel, Teresa R. Zembower, Frank Palella, Yashpal S. Kanwar, Shubhada N. Ahya

**Affiliations:** ^1^ Division of Infectious Diseases, Northwestern University, Feinberg School of Medicine, Department of Internal Medicine, Chicago, IL, USA; ^2^ Division of Pathology, Northwestern University, Feinberg School of Medicine, Department of Internal Medicine, Chicago, IL, USA; ^3^ Division of Nephrology, Northwestern University, Feinberg School of Medicine, Department of Internal Medicine, Chicago, IL, USA

**Keywords:** acute renal failure, highly active antiretroviral therapy (HAART), human immunodeficiency virus (HIV), tenofovir disoproxil fumarate (tenofovir, TDF)

## Abstract

Tenofovir is an acyclic nucleotide analogue reverse transcriptase inhibitor that is commonly prescribed as part of a highly active antiretroviral therapy (HAART) regimen in HIV-infected patients. Although it is generally well tolerated, renal insufficiency has been associated with its use. We report a biopsy-proven case of acute renal failure that developed within weeks of initiating a HAART regimen containing tenofovir, and review the literature with specific attention to cases of renal failure occurring within 8 weeks of tenofovir initiation. Our patient developed renal insufficiency within 3 weeks of initiating tenofovir-containing HAART and overt renal failure was noted within 5 weeks. Renal biopsy demonstrated histopathologic changes suggestive of HIV nephropathy, despite normal baseline serum creatinine values. Thirty additional cases of tenofovir-associated renal failure have been reported. In the majority (n = 22, 73%), renal failure occurred months after initiating therapy (range: 5–26 months). However, in a significant subset (n = 8, 27%), renal failure occurred within 8 weeks of treatment initiation. Our data suggest that some patients are at risk for developing renal failure within weeks of tenofovir initiation. Thorough evaluation of renal function should be undertaken before prescription of tenofovir-containing HAART. For those in whom subclinical renal disease is discerned, added vigilance when monitoring renal function may be warranted.

## INTRODUCTION

We report a case of acute renal failure in an HIV-infected patient receiving tenofovir. Tenofovir is an acyclic nucleotide analogue reverse transcriptase inhibitor with activity against HIV and hepatitis B. It has been used in combination highly active antiretroviral therapy (HAART) regimens for the treatment of HIV infection since October 2001[1,2,3]. In patients with normal renal function, it is administered once daily as an orally available tablet and offers a convenient therapeutic option as part of a once daily

HAART regimen[4]. Tenofovir is generally well tolerated, although acute renal failure has been identified as a potential adverse reaction. We review the existing literature and highlight cases of early-onset renal failure that were noted within 8 weeks of drug initiation.

## METHODS

A retrospective review was undertaken using PubMed to identify all cases of tenofovir-associated renal failure reported in the English language. All cases were analyzed for clinical characteristics and for pathologic characteristics when biopsy results were reported. Summary statistics and *p*-values using Fisher's exact test were conducted using Microsoft Excel® 2003(Microsoft Corporation, Redmond, WA).

## RESULTS

### Case Report

A 44-year-old African-American man with AIDS presented in June 2003 with a 2-week history of malaise and decreased urine output. At the time of tenofovir initiation, his $CD4^+$ count was 57 cells/$mm^3$ (7%), HIV-1 plasma RNA level was 1339 copies/m, and serum creatinine was at baseline 0.9–1.1 mg/dl. His past medical history was significant for chronic, but stable, right-sided hydronephrosis, anemia, recurrent bacterial pneumonia, oral candidiasis, Varicella-zoster virus, and disseminated mycobacterium avium complex (MAC) infections. He had been followed closely for mild, stable, right-sided functional hydronephrosis attributed to lymphadenopathy from disseminated MAC complex for 1 year.

He was receiving total parenteral nutrition for wasting. He was homosexual, and had a prior history of alcohol abuse and no history of intravenous drug use**.** The patient was diagnosed with HIV in 1987. He was initially treated with AZT, but then self-discontinued. Ten years later, he was treated with d4T, 3TC, and nelfinavir. Two years prior to this presentation, he was switched to d4T, ddI, and lopinavir/ ritonavir. One month prior to his presentation with renal failure, he was started on his current HAART regimen consisting of lamivudine 150 mg twice daily, efavirenz 600 mg once daily, tenofovir 300 mg once daily, and abacavir. Abacavir had been discontinued 2 weeks earlier with suspicion of a hypersensitivity reaction and, at that time, his serum creatinine (Cr) was 1.9 mg/dl. A renal ultrasound was repeated then, which demonstrated stable and mild right hydronephrosis without change from a previous study done 6 months earlier.

Laboratory evaluation 5 weeks after tenofovir initiation revealed a sodium 137 meq/l, potassium 4.3 meq/l, chloride 110 meq/l, bicarbonate 21 meq/l, blood urea nitrogen (BUN) level of 30 mg/dl (normal 0–20 mg/dl), and serum Cr of 3.7 mg/dl. The patient was aggressively hydrated intravenously overnight. Serum Cr on the following day was 4.1 mg/dl.

The HAART regimen was held. Physical examination was notable for a temperature of 103°F. Additional laboratory evaluation was notable for a serum hemoglobin of 10.0 gm/dl (normal 13.0–17.5 gm/dl) and a serum platelet count of 128 k/UL (normal 140–390 k/UL). Liver function studies revealed an elevated alkaline phosphatase level of 342 u/L (normal 30–115 u/L), consistent with his prior values. This was attributed to his disseminated MAC infection. Blood cultures drawn at the time of admission grew *Serratia marcescens*, and treatment was initiated with intravenous cefotaxime 2 g every 8 h.

Urinalysis revealed 1+ proteinuria and an elevated leukocyte esterase. Microscopic urine examination was significant for eight WBCs and over 800 RBCs per high powered field. No casts were noted. Urine culture was negative. Urine chemistries were notable for sodium of 51 meq/l and a fractional excretion of sodium of 2.2%. A 24-h urine collection demonstrated 1.1 g of protein. However, additional acid-base and urine studies, such as assessment for glycosuria or phosphaturia, were not done.

Further evaluation revealed normal quantitative plasma anti-Sm, anti-RNP, anti-SSA and SSB, antihistone, anti-Scl-70 and Jo-1, myeloperoxidase and proteinase-3 antineutrophil cytoplasmic antibodies, and C3 and C4 complement levels. Antinuclear antibody was positive with a titer of 1:160; anti-DNA antibody was negative. Initial serum and urine protein electrophoresis studies (SPEP, UPEP) each demonstrated two suspicious restricted bands in the gamma region. The UPEP also was notable for a restricted band in the beta region; however, repeat testing 4 months later was unremarkable. Renal ultrasound revealed mild hydronephrosis of the right kidney measuring 12.4 cm and the left kidney 13.3 cm (normal 9–12 cm), unchanged from previous studies. Differential diagnoses included HIV nephropathy, acute tubular necrosis, infiltrative processes, and acute medication–related interstitial nephritis.

Renal biopsy was performed on hospital day 4 and revealed 10% sclerosed glomeruli and 15% of glomeruli with ischemic injury, including partial collapse of the capillary loops. Remaining glomeruli demonstrated mild mesangial proliferation, increased mesangial matrix, and thickened capillary loops. Moderate degenerative tubular changes with loss of 30% tubular mass, interstitial scarring, and scattered cellular infiltrates were also noted. There was no evidence of vasculitis. Immunofluorescence staining showed only mild to moderate reactivity of IgG and C3; no other deposits were observed. Electron microscopy (Fig. 1) revealed mild diffuse fusion of podocyte foot processes. Mild hypertrophy in the endothelial and mesangial cells was also seen, as were increases in the number of lysosomes within podocytes. Influx of polymorphonuclear cells and monocytes was also noted. These changes were interpreted as consistent with acute renal injury superimposed on chronic renal damage, likely due to HIV nephropathy.

The patient's serum Cr peaked at 4.8 mg/dl 8 weeks after tenofovir initiation. He did not require hemodialysis. His renal function slowly improved off of HAART. Within 2 months after hospital discharge, serum Cr returned to baseline. At that time, a new HAART regimen consisting of zidovudine, lamivudine, lopinavir-ritonavir, and efavirenz was initiated, and the serum Cr has remained stable at his baseline value (Fig. 2).

## Literature Review

Reports describing tenofovir-associated nephrotoxicity have recently been described. Two of the first such reports were described in 2002[5,6], and to date, 30 cases have been reported (Table 1)[5,6,7,8,9,10,11,12,13,14,15,16,17,18,19,20,21]. The mean age of affected patients was 46 years (range: 31–60 years). The average elapsed time from tenofovir initiation to renal dysfunction diagnosis was 33 weeks (median: 28 weeks, range: 3–104 weeks). Mean peak serum Cr level was 3.1 mg/dl (range: 0.9–7.8 mg/dl).

Although tenofovir-related renal failure is more likely to occur at least several months after tenofovir initiation, eight of the 30 reported cases (27%) manifested renal failure within 2 months (Table 1 and Fig. 3). The remaining 22 patients developed renal failure from 5 to 26 months after starting therapy. This analysis suggests there may be two subsets of patients who experience tenofovir-induced renal failure: a minority who develop disease early vs. the more common scenario of those who develop it later after at least several months of therapy[22].

Of note, patients in this series were more likely to have undergone a renal biopsy with early rather than late onset of renal failure[4/8 (50%) vs. 5/22 (23%), $p = 0.2$]. One person who did not undergo renal biopsy developed early renal failure requiring continuous venovenous hemofiltration and experienced fatal lactic acidosis coincident with the kidney disease (Case 6)[11]. None of the other patients required hemodialysis, and in all other cases, renal failure resolved with discontinuation of tenofovir.

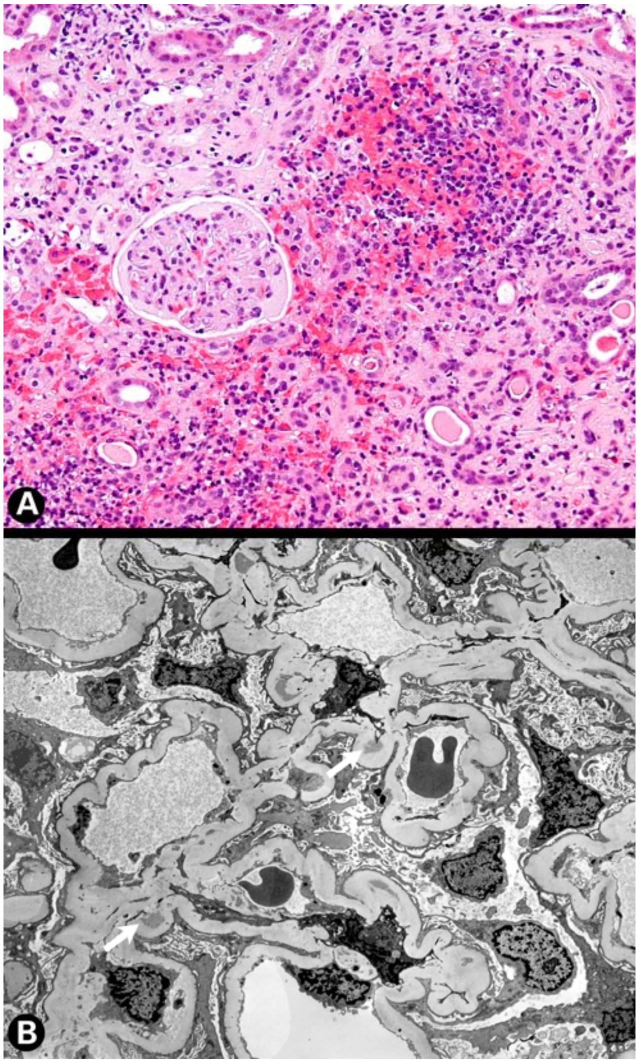

**FIGURE 1.** Renal biopsy of case patient 5 weeks after tenofovir initiation. (A) Renal biopsy section H & E stain showing focal and scattered cellular infiltrates. (B) Electron micrograph demonstrating mild diffusion of foot processes and moderate thickening with wrinkling of the glomerular basement membranes (arrow).

## DISCUSSION

Herein, we describe a case of acute renal failure consequent to tenofovir use in an HIV-infected patient noted 5 weeks after initiation of tenofovir-containing HAART. This case of biopsy-proven renal failure occurred despite baseline serum Cr values for this patient ranging from 0.8 to 1.1 mg/dl. While it is now clear that tenofovir use can result in mild reductions in renal function after at least several months of use in many individuals so treated, the prevalence and severity of nephrotoxicity observed does not appear to approach those seen with its' predecessor drugs, adefovir and cidofovir. Like adefovir and cidofovir, however, tenofovir can cause renal insufficiency through direct proximal tubular injury[23,24,25]. Renal failure from direct proximal tubular injury resulting in isolated proximal tubule absorption defects causing Fanconi-like syndrome or severe acute tubular necrosis has been well described with both of the latter two drugs[26].

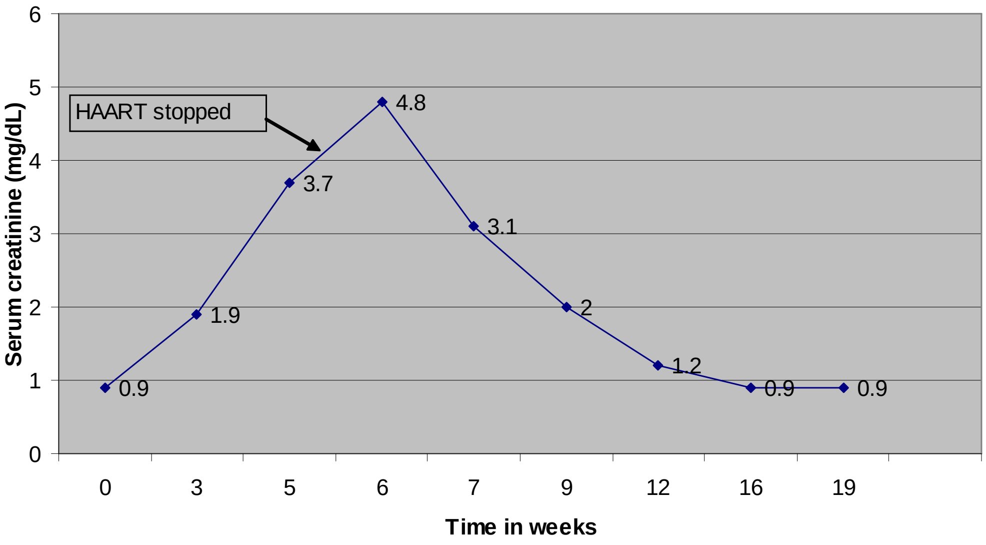

**FIGURE 2.** Renal function in patient receiving tenofovir-containing HAART.

Tenofovir, like adefovir and cidofovir, is excreted through the kidneys via glomerular filtration and tubular secretion. Hence, concomitant administration with medications also secreted by tubular cells via utilization of compensatory basolateral transporters (organic anion transporter-1, OAT-1) could interfere with tenofovir secretion, resulting in increased tenofovir blood levels. It is postulated that ritonavir-containing combination therapy may increase tenofovir levels by inhibiting the tubular apical protein transporter MRP-2 and hence block luminal secretion of the drug[21]. Concomitant use of these drugs raises the theoretical concern of enhanced tenofovir-associated nephrotoxicity. Recent data among persons simultaneously receiving tenofovir and ritonavir-boosted protease inhibitors, however, have not revealed increased nephrotoxicity in comparison with tenofovir recipients not receiving ritonavir-boosted regimens[27]. Our patient was not receiving concomitant nephrotoxic agents, and his renal function normalized once tenofovir was stopped.

Of note, our patient did not have evidence of significant renal impairment prior to receiving tenofovir; however, renal biopsy findings were reminiscent of early HIV-associated nephropathy as evidenced by ischemic collapse of the glomeruli and chronic tubulointerstitial disease. Our patient did not undergo the complete evaluation of renal function that is now recommended by the Infectious Diseases Society of America prior to beginning tenofovir therapy[22]. These recommendations state that renal function should be assessed at HIV diagnosis, and either annually for those without evidence of renal disease or semiannually for those receiving either indinavir or tenofovir. Additional assessments of renal function immediately prior to tenofovir initiation are not recommended at this time. If subclinical nephropathy exists, in this case demonstrated by renal biopsy, such patients may be predisposed to the more rapid onset of tenofovir-induced renal failure.

A limitation of this study is that the only information available is that which is included in the case reports. The impact of newer guidelines that recommend baseline and intermittent renal function assessment, including glomerular filtration rate determinations and assessments for microalbuminuria, will facilitate discernment of renal disease that is not detectable using only serum Cr values. However, the needed frequency of such evaluations for tenofovir recipients is unclear at present. Recent data suggest that tenofovir-associated renal failure occurs at a median duration of 43.2 weeks post-treatment initiation[25]. In contrast to this, our data suggest that a small, but significant, minority of tenofovir initiators will develop renal disease within several weeks of therapy initiation. We urge vigilance when monitoring renal function early after initiation of therapy among tenofovir recipients, especially those with evidence of subclinical renal disease.

**TABLE 1**
**Cases of Tenofovir-Associated Renal Failure Reported in the Literature**

| Case # [Ref.] | Age (Years) | Duration of Treatment with Tenofovir* | Concurrent HAART Regimen | Peak Cr Levels (mg/dl) | Renal Bx Findings |
|---|---|---|---|---|---|
| 1[26] | 55 | 3 weeks | Tenofovir, lamivudine, efavirenz | 5.7 | Biopsy not performed |
| 2[14] | 60 | 4 weeks | Ritonavir, atazanavir, didanosine, stavudine | 4 | Necrotic tubular lesions |
| 3[19] | 46 | 1 month | Didanosine, efavirenz | 1.8 | Biopsy not performed |
| 4[18] | 57 | 5 weeks | Abacavir, lamivudine | 4.3 | Tubulointerstitial nephropathy with primarily lymphocytic infiltrate |
| 5[5] | 44 | 6 weeks | Abacavir, didanosine | 6.2 | Acute tubular necrosis |
| 6[8] | 49 | 7 weeks | Didanosine, amprenavir, ritonavir | 7.6 | Biopsy not performed |
| 7[15] | 51 | 8 weeks | Lamivudine, stavudine, lopinavir/ritonavir | 3.5 | Interstitial infiltrate, focal atrophic changes, luminal ectasia, loss of brush border |
| 8[23] | 47 | 2 months | Saquinavir, abacavir, lopinavir/ritonavir, enfuvirtide | 2.3 | Biopsy not performed |
| 9[6] | 45 | 5 months | Didanosine, lopinavir/ritonavir | 2.2 | Vacuolation of proximal tubular epithelial cells and fading of the brush border |
| 10[21] | 39 | 5 months | Lamivudine, lopinavir/ritonavir | 6.6 | Biopsy not performed |
| 11[22] | 43 | 5 months | Lamivudine, lopinavir/ritonavir | Unknown | Biopsy not performed |
| 12[17] | 31 | 6 months | Didanosine, lamivudine, ritonavir, amprenavir, T20 | 1.7 | Bx not performed |
| 13[20] | 50 | 6 months | Stavudine, lamivudine, lopinavir/ritonavir | 5.1 | Severe ATN |
| 14[17] | 55 | 7 months | Abacavir, lopinavir/ritonavir | 7.8 | Severe ATN, interstitial fibroedema |
| 15[18] | 48 | 7 months | Lopinavir/ritonavir, lamivudine, efavirenz | 1 | Biopsy not performed |
| 16[13] | 38 | 7 months | Tenofovir, efavirenz, abacavir, amprenavir | 1.7 | Biopsy not performed |
| 17[24] | 54 | 8 months | Lamivudine, nevirapine, lopinavir/ritonavir | 3 | Biopsy not performed |
| 18[18] | 49 | 9 months | Didanosine, lamivudine, ritonavir, amprenavir | 1.1 | Biopsy not performed |
| 19[13] | 53 | 9 months | Tenofovir, abacavir, atazananvir | 1.8 | Biopsy not performed |

**TABLE 1 (continued)**

| Case # [Ref.] | Age (Years) | Duration of Treatment with Tenofovir* | Concurrent HAART Regimen | Peak Cr Levels (mg/dl) | Renal Bx Findings |
|---|---|---|---|---|---|
| 20[18] | 56 | 10 months | Lopinavir/ritonavir, lamivudine, efavirenz | 0.9 | Biopsy not performed |
| 21[17] | 42 | 11 months | Lamivudine, abacavir, lopinavir/ritonavir | 2.71 | Severe ATN, interstitial fibroedema |
| 22[18] | 35 | 11 months | Abacavir, lopinavir/ritonavir | 3.9 | Biopsy not performed |
| 23[25] | Unknown | 12 months | Ttenofovir, ritonavir, amprenavir, lopinavir, abacavir | 1.2 | Biopsy not performed |
| 24[25] | Unknown | 12 months | Tenofovir, ritonavir, amprenavir, lopinavir, didanosine, efavirenz | 1.3 | Biopsy not performed |
| 25[13] | 46 | 14 months | Tenofovir, lamivudine, zidovudine, saquinavir | 2.1 | Biopsy not performed |
| 26[13] | 45 | 15 months | Ttenofovir, delavirdine, didanosine, atazanavir | 2.6 | Acute Tubular Necrosis |
| 27[18] | 42 | 16 months | Abacavir, lamivudine, lopinavir/ritonavir | 1.5 | Biopsy not performed |
| 28[18] | 41 | 16 months | Abacavir, nevirapine | 1 | Biopsy not performed |
| 29[13] | 34 | 18 months | Tenofovir, abacavir, efavirenz, lopinavir/ritonavir | 1.6 | Biopsy not performed |
| 30[16] | 34 | 26 months | Lopinavir/ritonavir, lamivudine, didanosine | Unknown | Biopsy not reported |

* Duration of treatment is listed as it appears in the original case reports.

## CONCLUSIONS

In summary, we report a case of acute renal failure in an HIV-infected patient receiving tenofovir. It is used in combination HAART regimens for the treatment of HIV infection. While it is generally well tolerated, acute renal failure is a potential adverse reaction.

This case highlights the need for thorough evaluation of renal function prior to beginning tenofovir therapy. Evaluation should include a serum chemistry panel with electrolytes, BUN, Cr, calculation of Cr clearance, blood pressure determination for evidence of hypertension, and urinalysis to detect the presence of proteinuria and/or hematuria. For patients initiating tenofovir therapy, renal function may need to be checked as often as every month, as our case suggests that a subset of patients with underlying renal disease may be at particularly high risk of developing early renal failure as a result of tenofovir use.

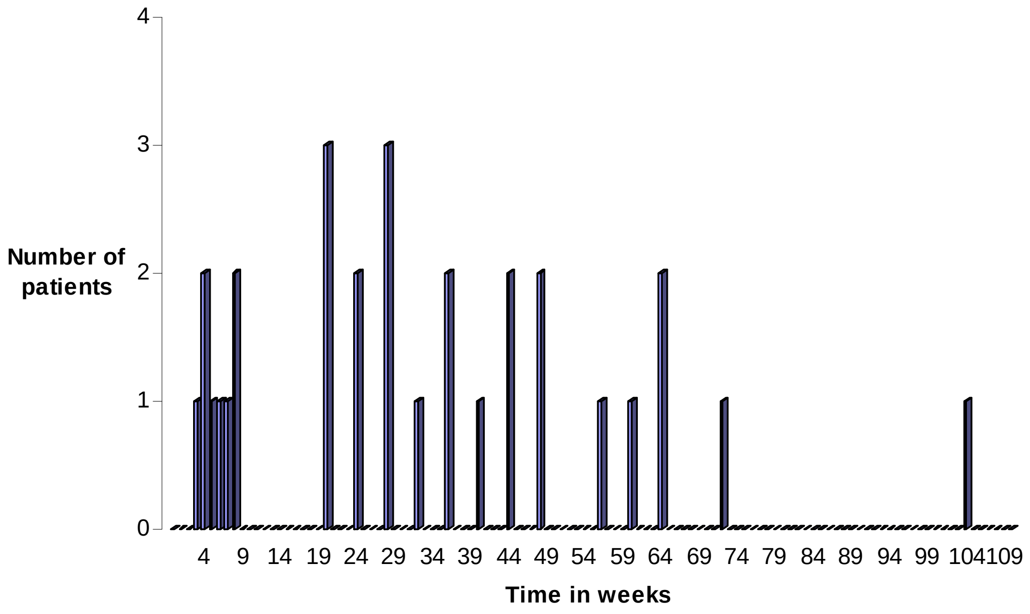

**FIGURE 3.** Spectrum of elapsed time between tenofovir initiation and detection of renal insufficiency in cases reported in the literature.

## ACKNOWLEDGMENTS

Financial support: this work was funded by Northwestern University Feinberg School of Medicine. Conflict of interest: Dr. Palella is on the Speakers Bureau for Gilead Sciences. Dr. Patel, Dr. Zembower, Dr. Kanwar, and Dr. Ahya do not have conflict of interest.

## References

1. Schooley, R., Ruane, P., Myers, R., et al. (2002) Tenofovir DF in antiretroviral-experienced patients: results from a 48-week, randomized, double-blind study. *AIDS* **16,** 1257–1263.
2. Barditch-Crovo, P., Deeks, S., Collier, A., et al. (2001) Phase I/II trial of the pharmacokinetics, safety, and antiretroviral activity of tenofovir disoproxil fumarate in human immunodeficiency virus-infected adults. *Antimicrob. Agents Chemother*. **45(10),** 2733–2739.
3. Margot, N., Isaacson, E., McGowan, I., Cheng, A., Schooley, R., and Miller, M. (2002) Genotypic and phenotypic analyses of HIV-1 in antiretroviral-experienced patients treated with tenofovir DF. *AIDS* **16,** 1227–1235.
4. (2002) *PDR 2003 HIV Treatment Manual*. 1st ed. pp. 329–336.
5. Coca, S. and Perazella, M. (2002) Rapid communication: acute renal failure associated with tenofovir: evidence of drug-induced nephrotoxicity. *Am. J. Med. Sci.* **324(6),** 342–344.
6. Verhelst, D., Monge, M., Meynard, J.L., et al. (2002) Fanconi syndrome and renal failure induced by tenofovir: a first case report. *Am. J. Kidney Dis.* **40,** 1331–1333.
7. Hansen, A.B., Mathiesen, S., and Gerstoft, J. (2004) Severe metabolic acidosis and renal failure in an HIV-1 patient receiving tenofovir. *Scand. J. Infect. Dis.* **36(5),** 389–392.
8. Creput, C., Gonzalez-Canali, G., Hill, G., Piketty, C., Kazatchkine, M., and Nochy, D. (2003) Renal lesions in HIV-1 positive patient treated with tenofovir. *AIDS* **17(6),** 935–937.
9. Barrios, A., Garcia-Benayas, T., Gonzalez-Lahoz, J., and Soriano, V. (2004) Tenofovir-related nephrotoxicity in HIV infected patients. *AIDS* **18**, 960–963.
10. Peyrière, H., Reynes, J., Rouanet, I., et al. (2004) Renal tubular dysfunction associated with tenofovir therapy: report of 7 cases. *J. Acquir. Immune Defic. Syndr*. **35,** 269–273.
11. Murphy, M., O'Hearn, M., and Chou, S. (2003) Fatal lactic acidosis and acute renal failure after addition of tenofovir to an antiretroviral regimen containing didanosine. *Clin. Infect. Dis*. **36,** 1082–1085.
12. Schaaf, B., Aries, S.P., Kramme, E., Steinhoff, J., and Dalhoff, K. (2003) Acute renal failure associated with tenofovir treatment in a patient with acquired immunodeficiency syndrome. *Clin. Infect. Dis*. **37**, e41–43.

13. Callens, S., De Roo, A., and Colebunders, R. (2003) Letter to the Editor - Fanconi-like syndrome and rhabdomyolysis in a person with HIV infection on highly active antiretroviral therapy including tenofovir. *J. Infect*. **47**, 262–263.
14. Gaspar, G., Monereo, A., García-Reyne, A., and Guzmán, M. (2004) Fanconi syndrome and acute renal failure in a patient treated with tenofovir: a call for caution. *AIDS* **18**, 351–352.
15. Earle, K.E., Seneviratne, T., Shaker, J., and Shoback, D. (2004) Fanconi syndrome in HIV+ adults: report of 3 cases and literature review. *J. Bone Miner. Res.* **19,** 714–721.
16. Karras, A., Lafaurie, M., Furco, A., et al. (2003) Tenofovir-related nephrotoxicity in human immunodeficiency virus-infected patients: three cases of renal failure, Fanconi syndrome, and nephrogenic diabetes insipidus. *Clin. Infect. Dis.* **36**, 1070–1073.
17. Lee, J.C. and Marosok, R.D. (2003) Acute tubular necrosis in a patient receiving tenofovir. *AIDS* **17**, 2543–2544.
18. Rifkin, B.S. and Perazella, M.A. (2004) Tenofovir associated nephrotoxicity: Fanconi syndrome and renal failure. *Am. J. Med*. **117,** 282–284.
19. James, C.W., Steinhaus, M.C., Szabo, S., and Dressler, R.M. (2004) Tenofovir-related nephrotoxicity: case report and review of the literature. *Pharmacotherapy* **24,** 415–418.
20. Breton, G., Alexandre, M., Duval, X., et al. (2004) Tubulopathy consecutive to tenofovir-containing antiretroviral therapy in two patients infected with human immunodeficiency virus-1. *Scand. J. Infect. Dis*. **36(6–7),** 527–528.
21. Rollot, F., Nazal, E.M., Chauvelot-Moachon, L., et al. (2003) Tenofovir-related Fanconi syndrome with nephrogenic diabetes insipidus in a patient with acquired immunodeficiency syndrome: the role of lopinavir-ritonavir-didanosine. *Clin. Infect. Dis.* **37,** e174–176.
22. Gupta, S.K., Eustace, J.A., Winston, J.A., et al. (2005) Guidelines for the management of chronic kidney disease in HIV-infected patients: recommendations of the HIV Medical Association of the Infectious Disease Society of America. *Clin. Infect. Dis.* **40,** 1559–1585.
23. U.S. Food and Drug Administration (2003) FDA Report: Background Package for NDA: VIREAD (tenofovir disoproxil fumarate). Available at: http://www.fda.gov/cder/approval/v.htm.
24. Becker, S., Balu, R., and Fusco, J. (2005) Beyond serum creatinine: identification of renal insufficiency using glomerular filtration: implications for clinical research and care. In Program and Abstracts of the12$^{th}$ Conference on Retroviruses and Opportunistic Infections (Boston). Foundation for Retrovirology and Human Health, Alexandria, VA. p. 819.
25. Gallant, J., Parish, M., Keruly, J., and Moore, R. (2005) Decline in renal function associated with tenofovir DF compared with nucleoside reverse transcriptase inhibitor treatment. In Program and Abstracts of the12$^{th}$ Conference on Retroviruses and Opportunistic Infections (Boston). Foundation for Retrovirology and Human Health, Alexandria, VA. p. 820.
26. Perazella, M. (2000) Acute renal failure in HIV-infected patients: a brief review of common causes. *Am. J. Med. Sci*. **319(6),** 385–391.
27. Gallant, J.E., Parish, M.A., Keruly, J.C., and Moore, R.D. (2005) Changes in Renal Function in Patients Treated with Tenofovir DF (TDF Compared to Nucleoside Reverse Transcriptase Inhibitors (NRTI's). The 3rd IAS Conference on HIV Pathogenesis and Treatment, July, Rio de Janiero, Brazil. Abstract no. TuPe2.3C18.
